# Agronomical valorization of eluates from the industrial production of microorganisms: Chemical, microbiological, and ecotoxicological assessment of a novel putative biostimulant

**DOI:** 10.3389/fpls.2022.907349

**Published:** 2022-07-22

**Authors:** Gabriele Bellotti, Eren Taskin, Maria Chiara Guerrieri, Gian Maria Beone, Cristina Menta, Sara Remelli, Fabrizio Bandini, Vincenzo Tabaglio, Andrea Fiorini, Federico Capra, Rossella Bortolaso, Simone Sello, Cristina Sudiro, Pier Sandro Cocconcelli, Francesco Vuolo, Edoardo Puglisi

**Affiliations:** ^1^Department for Sustainable Food Process (DiSTAS), Faculty of Agriculture, Food and Environmental Sciences, Università Cattolica del Sacro Cuore, Piacenza, Italy; ^2^Department of Chemistry, Life Sciences and Environmental Sustainability, University of Parma, Parma, Italy; ^3^ARPAE-Laboratorio Multisito di Ravenna, Ravenna, Italy; ^4^Department of Sustainable Crop Production (DI.PRO.VE.S.), Faculty of Agriculture, Food and Environmental Sciences, Università Cattolica del Sacro Cuore, Piacenza, Italy; ^5^LANDLAB S.r.l., Quinto Vicentino, Italy; ^6^SACCO S.r.l., Cadorago, Italy

**Keywords:** soil biostimulant, protein hydrolysate, HTS, soil biodiversity, soil bacteria, soil fungi, circular economy, soil fertility

## Abstract

Plant Biostimulants (BSs) are a valid supplement to be considered for the integration of conventional fertilization practices. Research in the BS field keeps providing alternative products of various origin, which can be employed in organic and conventional agriculture. In this study, we investigated the biostimulant activity of the eluate obtained as a by-product from the industrial production of lactic acid bacteria on bare agricultural soil. Eluates utilization is in line with the circular economy principle, creating economical value for an industrial waste product. The research focused on the study of physical, chemical, biochemical, and microbiological changes occurring in agricultural soil treated with the biowaste eluate, applied at three different dosages. The final aim was to demonstrate if, and to what extent, the application of the eluate improved soil quality parameters and enhanced the presence of beneficial soil-borne microbial communities. Results indicate that a single application at the two lower dosages does not have a pronounced effect on the soil chemical parameters tested, and neither on the biochemical proprieties. Only the higher dosage applied reported an improvement in the enzymatic activities of β-glucosidase and urease and in the chemical composition, showing a higher content of total, nitric and ammonia N, total K, and higher humification rate. On the other hand, microbial communities were strongly influenced at all dosages, showing a decrease in the bacterial biodiversity and an increase in the fungal biodiversity. Bioinformatic analysis revealed that some Operative Taxonomic Units (OTUs) promoted by the eluate application, belong to known plant growth promoting microbes. Some other OTUs, negatively influenced were attributed to known plant pathogens, mainly *Fusarium* spp. Finally, the ecotoxicological parameters were also determined and allowed to establish that no toxic effect occurred upon eluate applications onto soil.

## Introduction

The use of Plant Biostimulants (BSs) has increased in recent times in parallel with the awareness of the environmental impact of agriculture. BS represent a valid input that can supplement conventional fertilization on crops. Through BS application, conventional fertilizers are reduced, sustaining plant nutrition, and production nevertheless, while having a lower carbon footprint and higher eco-sustainability. Thus, BS can contribute to the reduction in chemical inputs in the agriculture system (de Pascale et al., 2017). According to the Regulation (EU) 2019/1009, products are considered BS when, regardless of their nutrient content, explicate at least one of the following beneficial activities: (i) mobilization of soil nutrients otherwise unavailable (ii) increase the efficiency of plant nutrient utilization, (iii) increase plant tolerance to abiotic stress factors, and (iii) improve plant quality traits (Rouphael and Colla, 2020). BS can be applied either at the foliar level, or on soil, in order to improve their nutritional level regardless of their nutrients’ content (García-García et al., 2020). In contrast to chemical fertilizers, which directly supply the soil with nutrients ready to be adsorbed by plants, BS foster the biogeochemical processes that are naturally occurring in soil (Hellequin et al., 2020). Furthermore, the application of BS can manipulate abundance and composition of soil-borne microorganisms, and this is sought especially when the number of saprophyte microorganisms increase, possibly leading to a higher formation of organic matter and increase availability of nutrients (Xun et al., 2016). The ability of BS to modify microbial activities can be attributed to their content of bioactive compounds such as phytohormones, organic/humic acids, and small peptides. Controlling the soil microbiome composition can help the sustainment of soil health, especially under sub-optimal conditions (Paul et al., 2019), so that crop plants can thrive and increase their production quality and quantity; all while providing a less costly, environmentally friendly, and more sustainable solution than synthetic fertilizers (Tejada et al., 2011).

Among the BS classes, protein hydrolysates (PHs) represent a major category. PHs derive from physico/chemical/enzymatic hydrolysis of by-product obtained from animal productions (e.g., collagen, blood) or from plant productions (legume seeds, alfalfa hay; Colla et al., 2015). Less commonly, also by-product from the dairy factories have been tested as biostimulants and their efficacy was demonstrated, but their preparation before usage requires some extra steps such as removal of NaCl (Caballero et al., 2020). As far as we know, no studies have investigated the effect of spent microbial eluates on soil proprieties. We tested the eluate obtained from the industrial production of lactic acid bacteria (LAB) which are mainly used for food and feed purposes. LAB production requires the use of batch fermentations, carried under strictly controlled conditions. The medium used for growing LAB is abundantly rich in macro- and micro-nutrients, and once cells are harvested into smaller volumes the remaining media is usually discarded as a waste product and undergo disposal (Mayra-Makinen and Bigret, 2004). The supernatant, or eluate, potentially carries prebiotic activity toward soil microorganisms at the rhizosphere level (Rodríguez-Morgado et al., 2017) and contains bioactive compounds such as polyamine which are related to plant stress response and antimicrobial compounds (Lamont et al., 2017). Analysis on the chemical composition of the eluate revealed its similarity in aminoacidic profile to other known biostimulants on the market, indeed, in accordance with du Jardin (2012) the eluate falls under the category of “N-containing substances.”

By-products obtained from the anaerobic fermentation of microorganisms may contain humic-like substances which can enhance soil fertility and phytohormones which are useful for plant health (Jorobekova and Kydralieva, 2019). However, few studies have investigate the impact of a plant biostimulant on bare soil microbial communities (Hellequin et al., 2020). Some authors tested the biostimulant effect of microbial eluates obtained from the dairy sector (Lamont et al., 2017; Caballero et al., 2020), but the preparation of such products requires some extra steps such as the removal of NaCl.

This paper will describe the methods adopted for the assessment of a putative biostimulant focusing on the chemical, biochemical, microbiological changes which occurred on bare soil after different applications of the eluate. The microbiological assessment was performed using High-Throughput Sequencing (HTS) technology and aimed to determine whether the changes in the microbial composition led to an increase/decrease in beneficial microorganisms or pathogens. Finally, ecotoxicology tests were carried out to demonstrate the eluate safety and whether its applications on soil can be linked to any toxic effects. We hypothesized that the eluate application can improve soil physico-chemical, biochemical features, and lead to a higher presence of beneficial microorganisms in soil and therefore display a biostimulant effect.

## Materials and methods

### Eluate description

The main characteristics of the eluate were provided by the producing company Sacco S.r.l. and comprised a minimal amount of nutrients N-P-K (4.5–1-0.5% w/w), a concentration of lactic acid >15% and micronutrients in traces. The end-of-fermentation broth from the LAB production is further harvested and concentrated under vacuum through heating at 95°C, to recover 90% of the water. This procedure is described and protected by the patent No. PCT/EP2021/080974.

This procedure guarantees the microbial and biochemical stability of the eluate, which maintain its integrity and composition for over 18 months at room temperature. This feature allows a better handling and conservation of the product for a very long-term use for the farmers and distributors. Finally, the treatment keeps the product in a liquid state, with a density of 1.11 g/l.

### Soil preparation and physico-chemical analyses

Pots of 1 L volume were filled with 1.5 kg of a sandy-soil mix (70–30%). Subsequently, the soil was treated with 80 ml of a solution containing the eluate at four different concentrations: 0 (control group), 2 g L-1 (T1), 4 g L-1 (T2), and 40 g L-1 (T3). Each thesis was tested preparing four biological replicates corresponding to four different pots. The substrate was then mixed thoroughly before sampling a total of 16 soil samples.

The soils were oven dried at 40°C to a constant weight, disaggregated to pass through a 2-mm sieve and homogenized. The soil proprieties were determined using standard methods (Ministero delle Politiche Agricole, 1999). The pH was measured in a 1:2.5 soil/water suspension. The cation exchange capacity (CEC) was measured after soil treatment with a barium chloride and triethanolamine solution with a pH of 8.1. The total limestone was obtained by gas-volumetric determination of CO2 by treating the sample with hydrochloric acid. The total C and N contents were determined by combustion in a furnace and using gas chromatographic analysis (vario MAX, Elementar, Langenselbold, Germany). Organic carbon was derived by subtracting limestone from the total C. The electrical conductivity in aqueous soil extracts were measured at a water/soil ratio 5:1. The extractable P was obtained by Olsen method while total P and K by inductively coupled plasma optical emission spectrometry (ICP-OES, Agilent mod. 5,800) after an acid mineralization in a microwave oven (CEM mod. MARS Xpress 5). The separation of non-humic fraction (NH) from the humic fraction (HA + FA) is obtained by solid phase adsorption chromatography (SPE) on polyvinylpyrrolidone resin. After having separated the humic acids by precipitation, the fraction of fulvic acids, is retained by the resin in an acidic environment. Subsequently, the adsorbed fulvic acids were eluted with a sodium hydroxide solution. Soil nitrate and ammonium were determined weighing 5 g of the soil in 50 ml plastic containers and 20 ml of K_2_SO_4_ 0.05 M was added. The containers were then placed under agitation at 220 rpm for 2 hours. Subsequently, the extract was filtered on Whatman paper no. 42 and was used for the analysis of both nitrate and ammonium. The nitrate concentration was determined by a double UV reading at 275 and 220 nm, after acidification of the samples with 1 M HCL (Kaneko et al., 2010; Mtolera and Dongli, 2018). For the determination of the ammonium concentration, on the other hand, the colorimetric reaction of Berthelot (Rhine et al., 1998) and absorbance reading at 760 nm was used. Both the analyses were performed in 96-well microplates in triplicate, with the use of a Biotek Synergy 2 spectrophotometer (Winooski, VT, United States).

### Soil ecotoxicology

Four different ecotoxicological tests were used: (i) acute toxicity test with *Daphnia magna* (method APAT IRSA CNR 8020—Man. 29: 2003—Volume third), (ii) light emission inhibition test with *Vibrio fischeri* (UNI EN ISO 11348-3: 2019), (iii) soil toxicity test with *Folsomia candida* ISO 11267 (ISO, 1999), and (iv) soil toxicity test with *Eisenia fetida* ISO 11268 (ISO, 1998). Their use in ecotoxicological tests reflects the need to highlight any toxic effects of the eluate at different levels of the aquatic ecosystem.

#### Acute toxicity test with *Daphnia magna* and light emission inhibition test with *Vibrio fischeri*

The soil samples were tested in the form of leachates due to their low solubility in water. For each soil sample 3 leachates were prepared, each with a different loading rate referred to the dry matter, using MilliQ water as leaching agent. The ratio chosen were: 1:4, 1:10, and 1:20. The values were chosen based on the indications provided by standards and methods for similar materials, in the absence of specific indications for this type of samples. The leaches were kept under stirring for 24 h in an orbital shaker at 100 rpm at the controlled temperature of 20–22°C. At the end, each suspension was left to settle for 15 min and was subsequently centrifuged at 3000 rpm to allow the separation of the solid and liquid phase. Only the aqueous phase was then taken for testing. The solutions obtained appeared partially transparent, with a minimal presence of fine/colloidal material in suspension, faintly colored in proportion to the applied loading rate. Before the tests were carried out, the aliquots of leached liquid were corrected with the salts required by the respective test method. The measured endpoints of the two tests reflect a different level of effect at the ecosystem level: in the assay with *D. magna* the immobilization of the organisms was measured, while in the test with *V. fischeri* the decrease in the natural light emission of the bacterium was evaluated.

##### Acute toxicity test with *Daphnia magna*

For each leached and for the negative control, 4 containers of 50 ml were prepared in which 5 daphnids were inserted. All the containers were incubated at 20 ± 1°C in an illuminated refrigerator thermostat at 300 lux for 48 h. At the end of the incubation, the immovable organisms were registered, and the percentage of immobilization was calculated according to the formula:


$$\%immobilization=1\text{0}0*\frac{no.immovableorganisms\;}{no.exposedorganisms}$$

##### Light emission inhibition test with *Vibrio fischeri*

The test was carried out using the MICROTOX M500 luminometer. The measure of the light emission was carried out after 5, 15, and 30 min of contact. The different reading times are indicative of the toxicity pathway and often to the nature to the contaminants present. For technical reasons it was not possible to test the sample at concentrations higher than 81.9% of the initial leachate. For each sample, a 1:2 dilution was prepared to determine the % EC50 value, which represents the percentage concentration of the test substance that determines a 50% effect in the organisms tested.

#### Toxicity test with *Folsomia candida*

The Collembola *F. candida* was used to test soil toxicity of the four different doses of eluate. Specimens came from laboratory cultures at Parma University. Growth, survival, and reproduction tests were carried out according to ISO 11267 (ISO, 1999). Specimens were maintained at 20 ± 2°C with 50–55% RH, on a diet of granulated dry yeast. To obtain synchronized cultures, adults from different breeding containers were transferred to new substrates, to stimulate egg release, and prevent juveniles originating from a single breeding line. All specimens used for the test were age-synchronized by removing eggs from the deposition cultures and once hatched, inserting juveniles into Petri dishes with moistened breeding substrate with a ratio of 8:1 (w/w) plaster of Paris and activated carbon powder.

For survival and reproduction tests, organisms (10 per Petri dish/replicate; three replicates per eluate dose) were added to each Petri dish using an exhauster, checking that none of the specimens had died during the process. Petri dishes, holding 30 g of wet test soil and approximately 2 mg of dry yeast as food source, were managed under the same conditions of the breeding cultures (aerated once a week and watered when water loss exceeded 2% of the initial water holding capacity—WHC) and incubated for 28 days. At the end of the test, adults and juveniles were euthanized by freezing. Petri dishes were filled with water and gently stirred with a spatula, allowing the animals to float on the water surface (floatation technique). A small amount of black ink, approximately 0.5 ml, was added to the water to increase the contrast between the water and the white Collembola. Then a digital picture was taken, and the number of surviving adults and new-born springtails were counted using image analysis software provided by ImageJ (version 1.53). The same procedure was applied to a substrate defined artificial soil in accordance with ISO 11267 (sphagnum peat/kaolinite clay/industrial quartz sand, 1:2:7 w/w; ISO, 1999).

#### Toxicity test with *Eisenia fetida*

The earthworm *E. fetida* (Oligochaeta: Lumbricidae) was used to test soil toxicity of the four different doses of eluate: 0 (control), 2, 4, and 40 g L-1. Sexually mature specimens were supplied by a worm breeding company. Survival and reproduction tests were carried out according to ISO 11268 (ISO, 1998). Earthworms (10 per container/replicate; three replicates per eluate dose) were rinsed with deionized water, patted dry with paper toweling, weighed, and introduced to each container. Test containers, filled with 500 g of wet testing soil (40–60% of the WHC), were maintained at 20 ± 2°C with 80–85% RH for 56 days. Approximately 5 g cattle manure was added to the surface of each container as food source. For 28 days, earthworms were fed weekly, and water was added when water loss >2% of the initial WHC. After 28 days, surviving earthworms were removed, counted, washed with deionized water, and weighed. Containers were then incubated for further 28 days under the previous conditions, except that food was provided only once, after adults’ removal. At the end of the test (56 days), juveniles were counted. As for *F. candida*, also for *E. fetida*, the same procedure was applied to an artificial substrate (artificial soil in accordance with ISO 11268: sphagnum peat/kaolinite clay/industrial quartz sand, 1:2:7 w/w; ISO, 1998).

### Soil enzymatic activities

The potential enzymatic activity of treated soil was used as a parameter to assess soil quality and was determined using *in vitro* enzymatic activity assays (Puglisi et al., 2006). The three enzymes’ activities measured were β-Glucosidase (β-GLU, EC 3.2.1.21), phosphatase (PHO, E.C. 3.1.3.2), and urease (URE, E.C. 3.5.1.5). Determination assays adopted were previously described in Eivazi and Tabatabai (1990) and Sannino and Gianfreda (2001). Briefly, for each enzyme the respective substrate was added to 1 g of soil. Then, after 1 h incubation at 37°C, in shaking conditions, samples were centrifuged and the supernatant was added to a colorimetric reactant specific for each enzymatic assay: urea, p-nitrophenyl-phosphate (PNP) and p-nitrophenyl-β-D-glucoside (PNG) respectively for testing URE, β-GLU, and PHO activities. The color was then read with a spectrophotometer reading β-GLU and PHO at 405 nm and URE at 690 nm using a 96 well micro plate. Comparing the samples optical measurements with the ones previously obtained with a calibration curve allowed to determine the enzymatic activities of treated soil. Measurements were expressed in μmol of substrate hydrolyzed at 37°C h−1 by 1 g of soil. Finally, the enzymatic activity values were used to calculate Alteration Index 3 (AI 3) using the following equation:


AI3=(7.87xβGLU)−(8.22xPHO)−(0.49xURE)

The index score was validated in Puglisi et al. (2006) and it is used for evaluating the alteration state of different soils according to the values of the most studied enzymes. The higher the AI 3 value, the more the soil can be considered altered. Negative values indicate lower levels of alteration in soil.

### Soil DNA amplification and high-throughput sequencing (HTS)

HTS was used to identify microbial changes upon the eluate application at different dosages. For each sample, 450–500 mg were weighted. The whole soil DNA was extracted using the FastDNA^™^ SPIN Kit for Soil (MP Bio, United States) according to the manufacturer’s protocol. Subsequently, 2 μl of extract were used to quantify the DNA extraction yield with the Quant-iT^™^ HS ds-DNA assay kit (Invitrogen, Paisley, United Kingdom) using a QuBit^™^ fluorometer. Moreover, 2 μl of extract were run on 1% agarose gel to verify the DNA quality was good before proceeding with the DNA amplification. The DNA extracted was then used to profile bacterial and fungal diversity in treated and untreated soil. The bacterial population was assessed by PCR amplification of the V3-V4 hypervariable regions of the 16S rRNA gene, using the universal primers 343f (5′-TACGGRAGGCAGCAG-3′), and 802r (5′-TACNVGGGTWTCTAATCC-3′; Misci et al., 2021). The PCR mix comprised 12.5 μl of Phusion Flash High-Fidelity Master Mix (Thermo Fisher Scientific, Inc., Waltham, MA, United States), 1.25 μl of each primer at the concentration of 10 μM, 1 ng of DNA template, and 8–9 μl of nuclease free water. The thermocycler program was set with an initial denaturation step at 94°C for 5 min, followed by 20 cycles of denaturation at 90°C for 30 s, annealing at 50°C for 30 s, extension at 72°C for 30 s, and a final extension at 72°C for 10 min. The fungal community was assessed by PCR amplification of the Internal Transcribed Spacer 1 (ITS1) region of ribosomal DNA (rDNA), using the universal primers ITS-1 (5′- TCCGTAGGTGAACCTGCGG-3′) and ITS-2 (5’-GCTGCGTTCTTCATCGATGC-3′; Bandini et al., 2020). The reaction mix had the following composition: 12.5 μl of Phusion Flash High-Fidelity Master Mix (Thermo Fisher Scientific, Inc., Waltham, MA, United States), 1.25 μl of each primer at the concentration of 10 μM, 1 ng of DNA template, and 8–9 μl of nuclease free water. The thermocycler was set as follow: initial denaturation at 94°C for 4 min, followed by 28 cycles of denaturation at 94°C for 30 s, annealing at 56°C for 30 s, an extension at 72°C for 1 min; and a final extension at 72°C for 7 min.

HTS method comprised the use of indexed forward primer, of which sequence was modified with a known 9 nucleic acid–base extension at the 5′ end, acting as a unique barcode. Tag primers enabled many samples to be sequenced in parallel without losing their origin. To reduce the possibility of anomalous PCR products, due to unspecific primers annealing caused by the 9 nucleic acid extension itself, a two-step PCR was performed as described in Berry et al. (2011), doing 20 PCR cycles with non-barcoded primers and then 10 cycles with barcoded primers. The final PCR products were quantified with QuBit^™^ fluorometer and then mixed in equal nanogram quantities in two separate pools, one for bacteria and one for fungi (30 ng of each product in each pool). The two pools were then purified with the solid phase reversible immobilization (SPRI) method Agencourt AMPure XP kit (Beckman Coulter, Italy) according to the manufacturer’s protocol, and sequenced by Fasteris S.A. (Geneva Switzerland), using the TruSeq DNA sample preparation kit (Illumina Inc., San Diego, CA) for amplicon library preparation. Sequencing was then performed with the MiSeq Illumina instrument (Illumina Inc., San Diego, CA) generating 300 bp paired-end reads.

Sequence data preparation and analyses began with the barcode demultiplexing, and base calling were processed with Illumina MiSeq Control Software version 2.3.0.3, RTA v1.18.42.0, and with CASAVA v1.8.2 (Bortolini et al., 2016). Raw reads were aligned, and the amplicon sequences were generated by the PANDAseq software (Masella et al., 2012), setting a minimum overlap of 30 bp between read pairs and allowing a maximum of 2 mismatches per sequence. Sequences were then demultiplexed according to the primer tag sequence using the software Fastx-toolkit.^1^ Chimeric sequences (homopolymers >10 bp) that did not align with the target sequence V3-V4 for bacteria and ITS1 for fungi, were considered outside the region of interest or belonging to non-targeted taxa and therefore removed using Mothur version 1.32.0 for bacteria sequences and UCHIME algorithm with the UNITE database for fungi sequences. The high-quality sequences obtained were processed with two different approaches, the operational taxonomic unit (OTU) and the taxonomy-based approach. For V3-V4 regions, OTUs and taxonomy matrixes were analyzed using Mothur V1.32.1 (Schloss et al., 2009). OTUs belonging to ITS1 amplicons were determined in UPARSE (Edgar, 2013) as no aligned databases are available for ITS1. Mothur was then set with a minimum length of 120 bp and no upper length limit due to ITS size variability.

Sequence data were submitted to the National Centre for Biotechnology Sequence Read Archive (NCBI-SRA) BioProject ID PRJNA827989.

### Statistic and bioinformatic analyses

Statistical difference of soil chemical analyses, enzymatic activities, alteration index and ecotoxicology data were determined with one-way analysis of variance (ANOVA), using the statistical software CoStat (Version 6,400, CoHort Software Monterey, CA, United States). The means were statistically compared by the honest significant differences Tukey’s HSD test at a 95% confidence level.

Statistical analyses on HTS data were performed using the software Mothur and R version 3.0.0^2^ supplemented with the Vegan package (Dixon, 2003). More details can be found in Vasileiadis et al. (2015). To visualize differences in the microbial populations at the corresponding eluate dosage, α-diversity analyses were based on the Shannon’s Index, the Observed Richness (S), the Simpson’s Diversity Index (D), and the Chao’s Index. The hierarchical clustering was prepared using the average linkage algorithm at different taxonomic levels. Principal component analysis (PCA) was performed to assess unconstrained sample groups, while canonical correspondence analyses (CCA) was used to assess the significance of different treatments on the analyzed diversity. Moreover, differential abundance of the most dominant OTUs accounting for at least 1% of reads for a given sample was determined using the Metastats algorithm (White et al., 2009), coupled with FDR test for means comparison (Paulson et al., 2011). Finally, the sequences belonging to significatively different OTUs, were individually submitted on taxonomy databases and identified at Genus or Species level. The databases were RDP (Ribosomal Database Project) and NCBI (National Center for Biotechnology Information) for bacterial OTUs and UNITE, RDP and Mycobank database for fungi OTUs.

## Results

### Soil physicochemical analysis

The addition of the eluate in soil pots corresponded to a total addition of N of 0.0072 g N/pot for T1, 0.0144 g N/pot for T2 and 0.144 g N/pot for T3. Soil physico-chemical results are summarized in Table 1. The analyses revealed a significative difference (*p* < 0.001) for pH values which resulted to be slightly higher for T1 and T2 corresponding at the eluate concentration of 2 and 4 g L^−1^. The exchangeable potassium was significantly higher at T3 (*p* < 0.001). The total nitrogen values increase in parallel with the eluate dosage but showed no significative difference according to the statistics, indicating a negligible effect in terms of total N supply of the treatments, and same for the C/N ratio. Nitric and ammonia nitrogen are significantly higher at T3, especially ammonia nitrogen which was almost 10 times greater compared to the control and the other thesis (*p* < 0.001), even though the single measurements showed great variability as showed from the standard deviation values in the table. Finally, the eluate addition to the soil caused a significant increase in humic substances, expressed through the HI and DH. Specifically, the T2 and T3 data show a very sharp change in the humic substance concentration compared to the control and the T1, pointing to a very pronounced effect of the eluate in modulating the richness of the soil fertility, organic matter degradation and water retention.

**Table 1:** Physicochemical characterization of the treated soils and the control.

	0 g L^−1^	2 g L^−1^	4 g L^−1^	40 g L^−1^
pH in water	8.36 (±0.1); b^**^	8.47 (±0.1); a^**^	8.47 (±0.1); a^**^	8.37 (±0.1); b^**^
CEC (cmol_(+)_ kg^−1^)	2.43 (±0.46)	2.89 (±0.31)	2.80 (±0.27)	2.60 (±0.26)
OC (g kg^−1^)	3.27 ± 0.32	3.27 ± 0.32	2.87 ± 0.25	3.57 (±0.15)
N_tot_ (g kg^−1^)	0.12 (±0.06); b^*^	0.14 (±0.06); b^*^	0.22 (±0.05); b^*^	0.26 (±0.06); a^*^
N nit (mg/kg)	27.0 (±4.4); ab^*^	21.15 (±6.5); b^*^	23.17 (± 4.38); b^*^	39.0 (±9.8); a^*^
N amm (mg/kg)	2.92 (±0.44); b^***^	3.01 (±0.08); b^***^	2.87 (±0.10); b^***^	55.55 (±21.53); a^***^
C/N	35 (±20)	27 (±12)	14 (±5)	14 (±4)
CaCO_3_ (g kg^−1^)	92 (±11)	89 (±7)	97 (±6)	94 (±2)
P_Olsen_ (mg kg^−1^)	10.2 (±0.4)	10.1 (±1.3)	9.6 (±0.7)	10.4 (±1.1)
K_exch_ (mg kg^−1^)	39.9 (±5.6); b^**^	36.5 (±1.6); b^**^	37.1 (±7.4); b^**^	56.6 (±6.6); a^**^
P_tot_ (mg kg-1)	349 (±25)	379 (±24)	336 (±27)	350 (±27)
K_tot_ (mg kg^−1^)	981 (±19)	1,039 (±44)	1,015 (±74)	1,016 (±33)
EC 5:1 (μS cm^−1^)	209 (±23)	147 (±42)	170 (±18)	219 (±34)
HI	0.33 (±0.06); b^*^	0.39 (±0.28); b^*^	1.33 (±0.65); a^*^	1.41 (±0.53); a^*^
DH%	76 (±4); a^*^	77 (±24); a^*^	46 (±15); b^*^	43 (±10); b^*^
HR%	28 (±9)	33 (±16)	21 (±5)	16 (±4)

ANOVA significant differences were indicated by *F* values (*p < 0.05, **p < 0.01, ***p < 0.005) for comparisons between rows and columns for treatments and time.

### Soil ecotoxicology

#### Acute toxicity test with *Daphnia magna*

The test complied with the validity criteria: (i) less than 10% of the control test organisms were found to be immobile after 48 h of exposure (Test result = 0%); (ii) less than 10% of the control test organisms showed signs of damage/stress after 48 h of exposure (Test result = 0%); (iii) the dissolved oxygen at the end of the test was ≥3 mg L^−1^ in the control and in the tested concentrations. The test results are reported in Table 2. The pH, conductivity and dissolved oxygen values were determined in the samples with a high percentage of immobilization: the values obtained were within the limits, therefore a direct effect of the eluate on the test organisms is excluded.

**Table 2:** Acute toxicity test results obtained after 48 h of exposure of *D. magna* at the soil leachates diluted 1:4, 1:10, and 1:20.

Dosage	1:4	1:10	1:20
0 g L^−1^	1.67 (±2.89); b^***^	0; b^***^	0; b^***^
2 g L^−1^	1.67 (±2.89); b^***^	0; b^***^	0; b^***^
4 g L^−1^	1.67 (±2.89); b^***^	0; b^***^	0; b^***^
40 g L^−1^	96.67 (±5.77); a^***^	76.77 (±15.28); a^***^	65 (±8.66); a^***^

Numbers express the % of immobilized specimens and show a significative difference at 40 g L^−1^ for all dilutions tested.

ANOVA significant differences were indicated by *F* values (*p < 0.05, **p < 0.01, ***p < 0.005) for comparisons between rows and columns for treatments and time.

#### Light emission inhibition test with *Vibrio fischeri*

As reported in Table 3, all the samples showed no toxicity after the light emission inhibition test; any effect measured is not statistically different from that shown by the control group 0 g L^−1^.

**Table 3:** *Vibrio fischeri* light emission test results obtained at three different reading times for three different dilutions of leachates.

Dosage	%EC50								
	1:4			1:10			1:20		
	5’	15’	30’	5’	15’	30’	5’	15’	30’
0 g L^−1^	>81.9	>81.9	>81.9	>81.9	>81.9	>81.9	>81.9	>81.9	>81.9
2 g L^−1^	>81.9	>81.9	>81.9	>81.9	>81.9	>81.9	>81.9	>81.9	>81.9
4 g L^−1^	>81.9	>81.9	>81.9	>81.9	>81.9	>81.9	>81.9	>81.9	>81.9
40 g L^−1^	>81.9	>81.9	>81.9	>81.9	>81.9	>81.9	>81.9	>81.9	>81.9

No significative difference found between the samples.

#### Toxicity tests with *Folsomia candida* and *Eisenia fetida*

**Figure 1:**
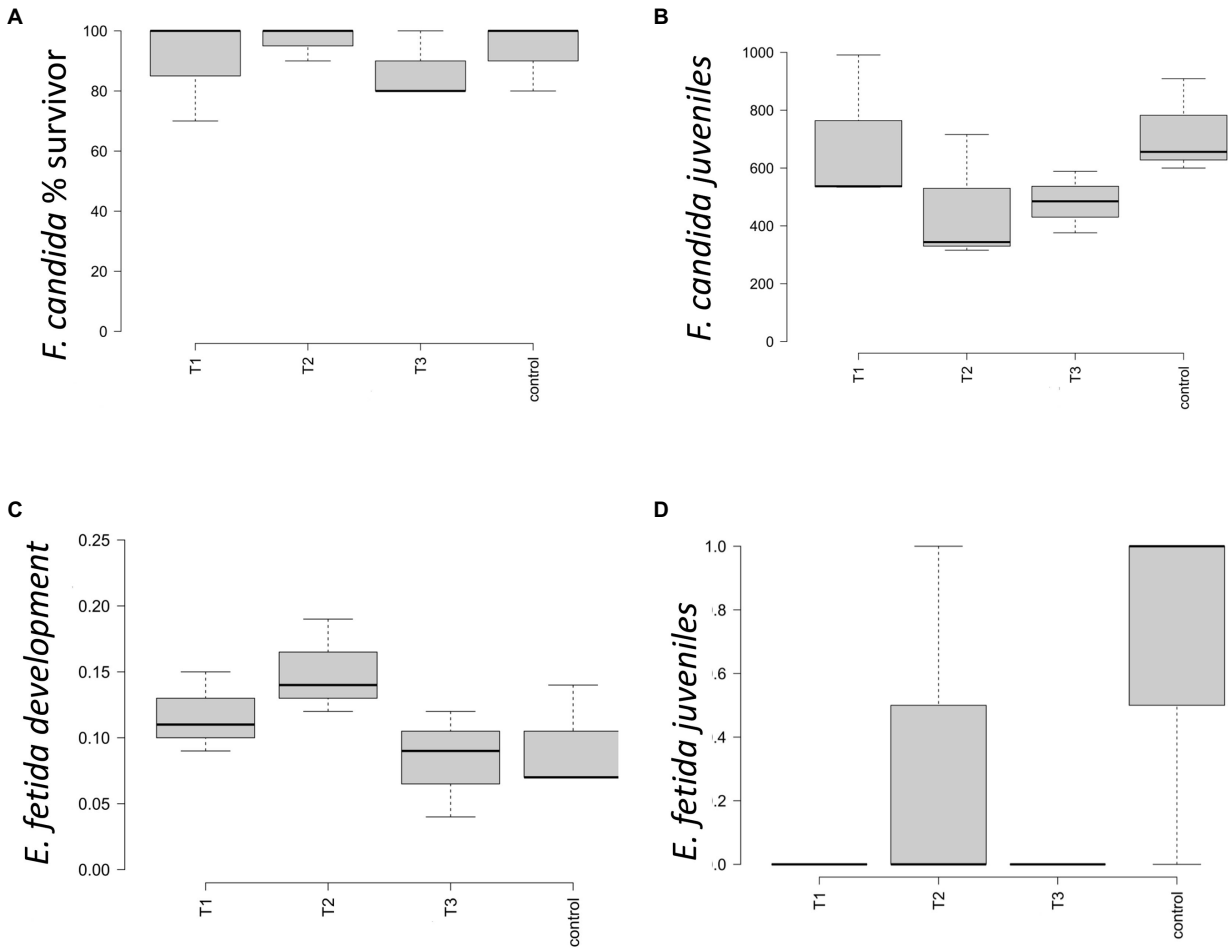
Ecotoxicity parameters measured for control = 0 g L^−1^, T1 = 2 g L^−1^, T2 = 4 g L^−1^, and T3 = 40 g L^−1^. The boxplot shows the average and standard error for **(A)** percentage of surviving springtail *F. candida*; **(B)** number of juveniles springtails *F. candida* born after 28  days; **(C)** average and standard error of the *Eisenia fetida* earthworms’ mass change (%); **(D)** number of *E. fetida* juveniles born after 28 and 56  days of test.

In the artificial soil prepared according to ISO 11267 and 11,268 (ISO, 1999,1998), the conditions of validity for the test were met both for *F. candida* and *E. fetida*. Survival of *F. candida* was comprised between 70 and 100% in the four conditions (Figure 1A), with the highest mean value of number of survivors in T2 and the lowest in T3; no differences were highlighted by statistical analysis. Reproduction showed a reduction in the number of juveniles in T2 and T3, but statistical analysis did not highlight differences between treatments (Figure 1B). At the end of the survival test (that is after 28 days), survival of *E. fetida* was 100% in all four conditions, so that no statistical differences between them were observed. *E. fetida* test reported no significative changes in the weight gain for the specimen introduced (Figure 1C) although an increasing trend can be observed in T2 and T3. Figure 1D showed no statistical differences between the number of juveniles born after 28 and 56 days.

### Enzymatic activities

Soil enzymatic activity measurements of β-GLU, PHO, and URE together with the alteration index score (AI3) are reported in Table 4. The eluate application significantly increased β-GLU activity at T3 compared to the control soil (*p* < 0.05). PHO activity was significantly lower in samples of T1 and T2 if compared with T3 (*p* < 0.05). However, T3 PHO values were not statistically different when compared to the control group. URE activity was significantly higher only at T3 (*p* < 0.01). Finally, the alteration index AI3 was calculated using the enzyme activities and showed a significative reduction (*p* < 0.05) at T3 compared to the lower dosages of T1 and T2 but showed no difference compared to the control.

**Table 4:** Enzyme activity measurements in different treatments.

Dosage	β-GLU	PHO	URE	AI3
0 g L^−1^	0.24 (±0.11); b^*^	2.44 (±0.21); ab^*^	2.70 (±0.10); b^**^	−19.53 (±2.27); ab^*^
2 g L^−1^	0.29 (±0.11); ab^*^	1.96 (±0.51); b^*^	3.43 (±0.14); b^**^	−15.49 (±5.09); a^*^
4 g L^−1^	0.34 (±0.04); ab^*^	2.16 (±0.19); b^*^	3.04 (±0.40); b^**^	−16.54 (±1.46); a^*^
40 g L^−1^	0.50 (±0.09); a^*^	3.40 (±0.60); a^*^	5.98 (±1.64); a^**^	−26.98 (±4.91); b^*^

Activities are expressed in μmol of substrate hydrolyzed at 37°C h^−1^ by 1 g of soil. Alteration index (AI3) was calculated as described in Puglisi et al. (2006).

ANOVA significant differences were indicated by *F* values (*p < 0.05, **p < 0.01, ***p < 0.005) for comparisons between rows and columns for treatments and time.

### Bacterial community structure

**Figure 2:**
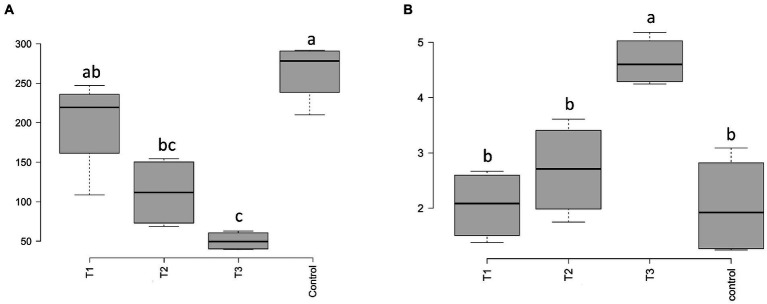
α-diversity values according to the Simpson’s D index for bacteria **(A)** and fungi **(B)** for each eluate dosage applied on bare soil (*p* < 0.001). Different letters indicate significant differences according to the one-way ANOVA test carried out by Tukey’s HSD (Honest Significant Differences).

**Figure 3:**
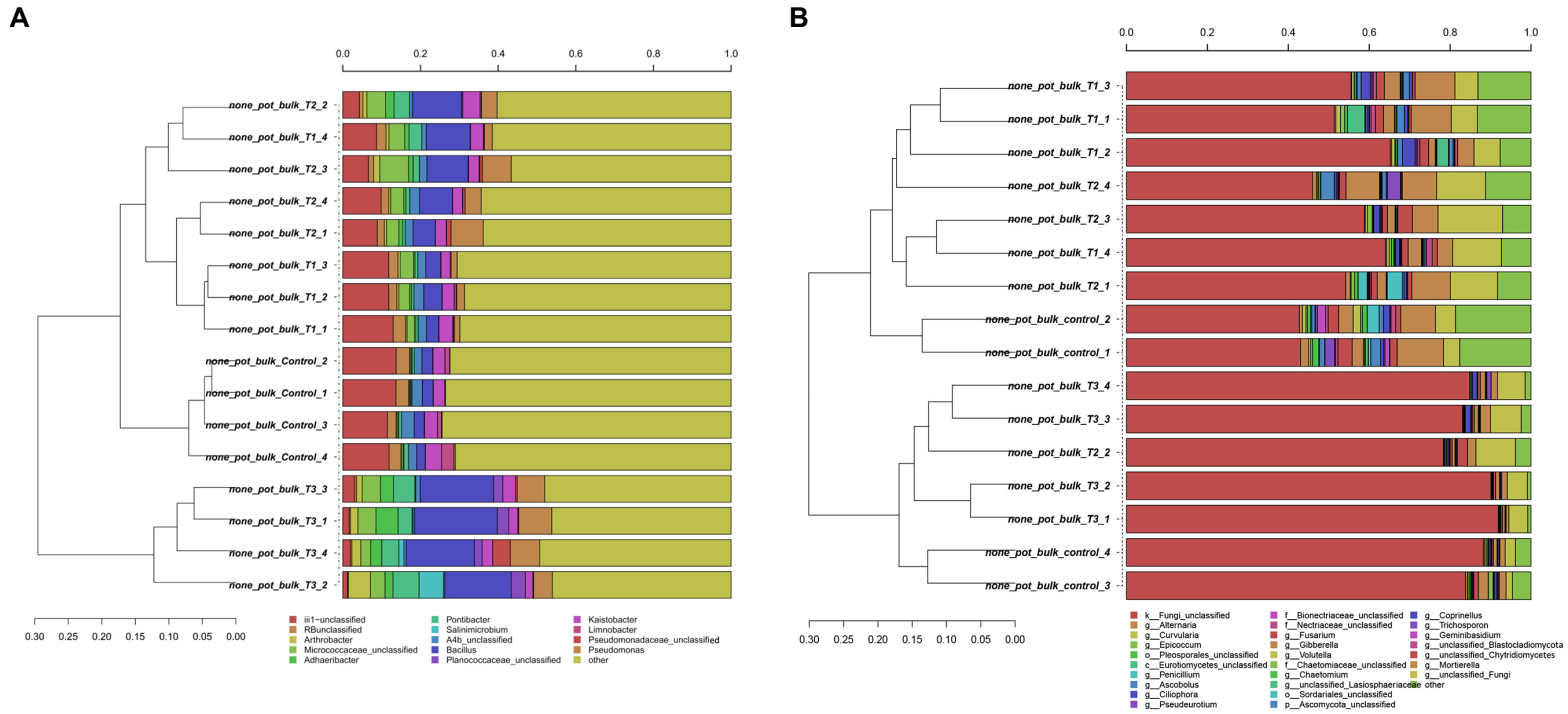
Hierarchical cluster classification at genus level for bacterial **(A)** and fungal **(B)** taxa contributing at more than 5% in at least one sample. Taxa contributing with a lower threshold are added to the “other” sequence group.

**Figure 4:**
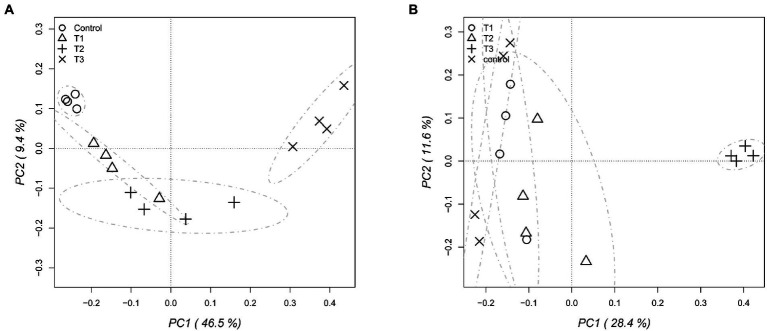
Principal component analysis (PCA) performed to assess the unconstrained grouping of the total bacterial **(A)** and fungal **(B)** OTUs, showing the relative abundance for each dose of eluate.

**Figure 5:**
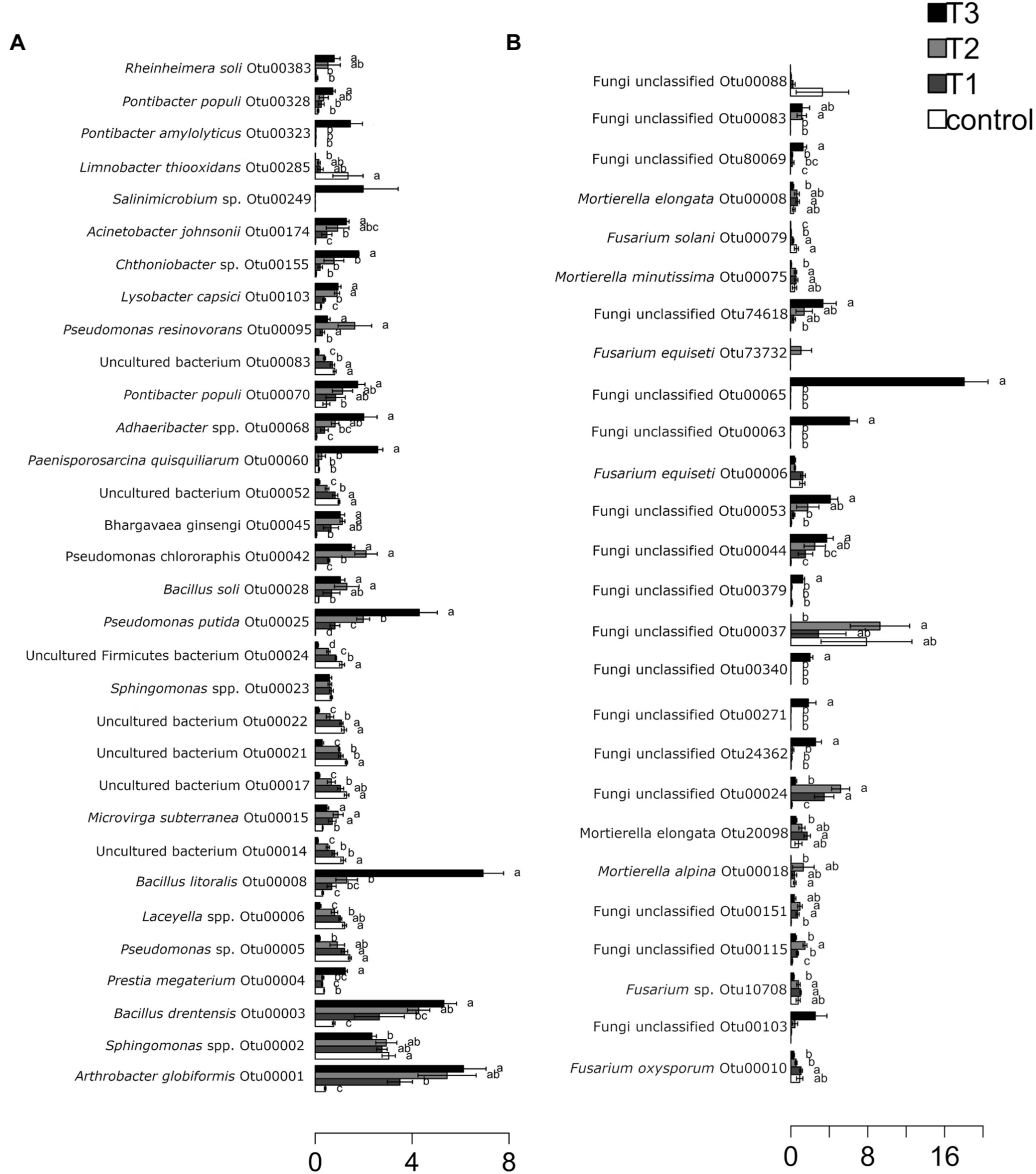
Metastats model output, indicating relative abundances of the 32 most abundant bacterial OTUs **(A)** and fungal OTUs **(B)** observed, comprising 95% of the bacterial diversity found in each treatment dose: control = 0 g/l, T1 = 2 g/l, T2 = 4 g/l, and T3 = 40 g/l. OTUs showing significant differences according to statistical analysis are highlighted with letters. The assigned genera or species was indicated where possible for each OTU.

After HTS analysis, 16S sequences were filtered and demultiplexed. Each sample counted a total of 13,500 high-quality reads and the related average coverage was 89.00%, thus indicating that a good part of the bacterial community diversity was covered by the analysis. Sequences were classified at 87.3% order 59.4% to family level, at 33.9% to genus level (data not shown). To estimate the values of α-diversity a multivariate analysis on the total OTU matrixes was performed. The output is shown in Figure 2A, represented by the Simpson’s Diversity index (D). D values were calculated according to the 16S amplicons variability and diversity. It shows that increments in the eluate dosage reduce the overall α-diversity values of the bacterial community. Especially T2 and T3 are significantly lower than the diversity found in the control group (*p* < 0.001). The taxonomy-based approach analyzed the OTUs using the hierarchical clustering coupled with the average linkage algorithm at different taxonomy levels. In Figure 3A is reported the bacterial hierarchical cluster output which shows that all the samples are mainly characterized by bacterial taxa contributing in less than 5% of the total and are therefore added in the group “other.” The genera positively influenced, which abundance rises along with the eluate application belong to *Bacillus*, *Pseudomonas,* unclassified *Micrococcaceae, Pontibacter*, *Adaheribacter* and *Arthrobacter*. Especially in T3 the genera *Bacillus*, *Adaheribacter* and *Pseudomonas* are abundant, showing a good affinity with the eluate. Compared to T2 and T3, T1 and the control are characterized by a major presence of unclassified bacteria/rhizobacteria, as well as taxa included in the group “other” indeed showing more diversity. Samples grouping in Figure 3A show a strong clustering pattern; the control group and T3 cluster separately showing composition difference. T2 and T1 are not entirely separate meaning that they share some features, yet differently compared to the control. The multivariate Principal Component Analysis (PCA) was performed on the OTUs’ relative abundance in each treatment to assess the unconstrained samples grouping. The output is presented in Figure 4A where the control and T3 clustered separately, while T1 and T2 shared more of their bacterial community and therefore they cluster. Finally, the Metastats model (used to identify any significant differences between the bacterial population of different samples) was used to highlight the most dominant OTUs which significatively differ between the treatments. A total of 32 bacterial OTUs were spot and identified at genus and, when possible, at species level with BLASTn analysis or RDP and NCBI databases. Upon scientific literature review some of these OTUs sequences were attributed to known PGPR and were positively influenced by the eluate application. Between them the most important were of *Arthrobacter globiformis*, *Bacillus drentensis*, *Priestia megaterium*, *Bacillus litoralis*, *Pseudomonas putida*. In Figure 5A is reported the taxonomic classification assigned to all the differentially abundant bacterial OTUs.

### Fungal community structure

After filtering and demultiplexing the ITS sequences, each sample counted a total of 25,000 reads. The related average coverage of fungi amplicons was 96.40%, thus indicating that most of the fungal diversity was covered by the analysis. The bioinformatic analysis repeated the same steps made for bacterial 16S sequences. The ITS multivariate analysis on the total OTU matrixes was performed to estimate α-diversity according to the Simpson’s Diversity index (D; Figure 2B). Fungal α-diversity resulted in a higher value for T3 (*p* < 0.001). No significative difference was found for the other theses, although an increasing trend can be observed comparing T1 and T2 treatments with the control. The taxonomy-based approach included hierarchical clustering coupled with the average linkage algorithm at different taxonomy levels, and the multivariate principal component analysis (PCA) was performed to assess the unconstrained grouping. Figure 3B shows the OTUs hierarchical clustering classified at the genus level. The vast majority of the OTUs belong to unclassified fungi in all the samples. The PCA output is reported in Figure 4B, and the graph shows that only T3 clusters separately, while the other treatments grouped all together with the control. Metastats script applied on the fungal OTUs highlighted the sequences which significatively differ between the treatments. These OTUs were identified at Genus and, when possible, at Species level with BLASTn analysis on NCBI, RDP and Mycobank databases. In total, 26 fungal OTUs were classified as differentially abundant. In Figure 5B the taxonomic classification of fungi corresponding to significantly different OTUs is reported and few OTUs positively influenced were attributed to known PGPF, mainly belonging to *Mortierella* Genus. Moreover, some OTUs of which abundance decreased were attributed to *Fusarium* spp.

## Discussion

According to the chemical analyses, the agricultural soil used can be classified as alkaline (Liu et al., 2016) and the eluate applied at 2 and 4 g L^−1^ yielded soil slightly more alkaline (*p* < 0.001). Despite the high content of lactic acid (>15%) in the eluate, the pH values of the control were not statistically different than the ones measured at 40 g L^−1^. Thus, the microbial changes which occurred are not explained by the soil pH which usually has a strong impact on microbial communities’ composition (Rousk et al., 2010). Furthermore, the physico-chemical analysis revealed that the exchangeable potassium was significantly higher at the eluate concentration of 40 g L^−1^ (*p* < 0.001) while no significant changes were found for the total nitrogen and potassium values. This indicates that the eluate application can increase the rate of available potassium in soil by mobilizing the unavailable fraction. According to this result the eluate complies with one of the four beneficial activities a BS can carry (du Jardin, 2012).

The soil potential enzymatic activities measured (β-GLU, PHO, and URE) were appropriate to determine the alteration state AI 3 of the treated soil (Puglisi et al., 2006). AI3 index was used as a comparative assessment tool for evaluating the eluate impact on the overall alteration state of soil. The values indicate an overall low alteration state of the soil in all samples. The alteration index calculated was significantly lower (*p* < 0.05) at the eluate dosage of 40 g L^−1^ compared to the other treatments, showing a lower alteration state at this dose (Taskin et al., 2021). Enzymatic assays and the alteration index suggest that the soil enzymatic activity was not strongly influenced by the eluate application although an increasing trend in the enzymatic activities can be observed for all the enzymes tested. Other studies employing BS from various sources of organic matter, have obtained stronger response in the enzymatic activity of treated soil (Bastida et al., 2008; Tejada et al., 2010, 2011). These studies, however, report an increase in enzymatic activities and soil chemical composition after 2–3 years period while in the present study the parameters were analyzed just after few days from the treatment. In general, soil enzyme activity can be attributed to either microbial activity or to the soil matrix. It is worth to mention that despite the shifts observed in α-diversity values, the enzymatic activities were not significantly altered, therefore it is possible to assume that most of the enzymatic activity measured are related to abiontic enzyme activity and are not related to microbial activities (Nannipieri et al., 2018).

Another factor likely to be explaining the altered enzymatic activity could be the increase in the humification index and the humification degree, registered in the samples treated with the highest concentrations of eluate. The significant enrichment of the humic substances over the total organic substances might act indirectly to stimulate the soil metabolic pathways of β-GLU and PHO, through the direct addition of the different nutrients of the eluate. On the contrary, the increase in the urease activity is instead directly proportioned only in the 40 g/l treated samples and could be explained by the highest concentration of Nitrogen supplied. Nevertheless, these results suggest that the lactic eluate can increase the soil fertility and enzymatic activity.

Ecotoxicology tests report no toxicity for the organisms *V. fischeri* and *F. candida*. Same for the test on *E. fetida* where all the specimens introduced in the four treatments survived the entire period of the survival test (28 days), so that no differences were observed between treatments. The highest value of earthworm development, in terms of mass change, was observed in 4 g L^−1^, with a weight gain that was almost twice those measured in 40 g L^−1^, but no statistical differences emerged. The number of juveniles was extremely low in each treatment, ranging between 0 and 1 specimens. Only in the control and in the 4 g L^−1^ juveniles were detected. Due to the exiguous juvenile production and the high variability intra-replicates, statistical analysis did not show differences. Acute toxicity test results show a very evident toxicity of the leachates obtained from the samples at the dosage of 40 g L^−1^. The percentage values of immobilization detected for these leachates indicate a high toxicity toward the organism *D. magna*. In the leachates at a concentration of 2, 4 g L^−1^ no toxicity was found; any effect detected is not statistically different from that shown by the negative control. The pH, conductivity and dissolved oxygen values were determined in the samples with a high percentage of immobilization: the values obtained were within the limits, therefore a direct effect of the eluate on the test organisms is excluded. The different effect detected in the *D. magna* tests can be simply explained by the nature of the contaminant/additive present in the eluate and by the respective sensitivity of the test organisms toward it (Nasser et al., 2020).

HTS of bacterial and fungal amplicons allowed to assess the microbial diversity across the different treatment dosages. The reduction in the α-diversity values measured for the bacterial community can be explained by a higher affinity of few microbial species to the eluate content of low molecular weight peptides, sugars, and organic acids. In general, some fast-growth rate Firmicutes and Actinobacteria are known to be strong competitors when put in environment rich in nitrogen and carbon nutrient sources (Meidute et al., 2008; Tejada et al., 2011; Ren et al., 2020). Those few bacterial species able to utilize this nutrient surplus developed and prevailed, while reducing the overall bacterial diversity. On the other hand, fungal α-diversity values increased along with the eluate concentration. A similar effect was obtained by Caballero et al. (2020) using a biostimulant obtained as dairy by-product which also contained lactic acid. Other studies carried on biostimulant proprieties of seaweed extract and an amino acids based biostimulants report a similar effect on the bacterial diversity, but they also show a reduction in the fungal diversity (Hellequin et al., 2020). However, the present analyses were performed after 4 days upon the treatment. It is therefore necessary to run this test also in different years, after recurrent use of the test eluate, and to verify its impact on the long-term (Hellequin et al., 2020).

The bacterial taxonomic hierarchical cluster and the related PCA graph highlight the difference in bacterial OTUs found in 40 g L^−1^ and in the control which cluster separately from the other samples. The main genera found to be thriving with increasing dosages of eluate were *Bacillus*, *Pseudomonas*, *Pontibacter, Adhaeribacter* and *Arthrobacter*. Especially *Bacillus* and *Pseudomonas* genera presence increases along with the dosage showing a good affinity with the eluate. These results are once again in line with HTS analysis carried by Caballero et al. (2020) where *Pseudomonadaceae*, *Bacillaceae*, and *Micrococcaceae* were dominant.

Using BLASTn on differentially abundant bacterial OTUs underlined the presence of beneficial bacteria. A very important outcome, regards OTU1, that was positively influenced by all the eluate dosages while it was barely present in the control. This OTU corresponds to *A. globiformis*, a soil-borne bacterium whose PGPR activities are known (Sharma et al., 2016). OTU3 was positively influenced by all the eluate dosages, especially by the highest dosage of 40 g L^−1^ indicating a great compatibility with the eluate. The OTU belongs to *B. drentensis*, soil-borne bacterium whose PGPR activity are also well characterized (Arikan and Pirlak, 2020). This species was also studied for the amelioration of salt stress tolerance in crop plants and for its bioremediation potential in mercury contaminated soil (Robas et al., 2021). OTU4 and OUT8, were both positively influenced by the eluate at the dosage of 40 g L^−1^, showed a good affinity with the eluate. The OTUs belongs, respectively, to *Priestia megaterium* one of the most studied PGPR (Kumar et al., 2011; Liang et al., 2011; Munjal et al., 2016) and to *B. litoralis*, a PGPR that is also able to establish a PGP synergistic action with arbuscular mycorrhizal fungi (Agnolucci et al., 2019). OTU25, that was positively influenced at all the eluate dosages. The OTU belongs to *P. putida*, a widely studied bacterium often carrying PGP proprieties, bioremediation capacity, biocontrol, and for induced systemic resistance in plants (Almaghrabi et al., 2013; Agisha et al., 2019; Asadullah and Javed, 2021). *P. putida* is ubiquitous in soil and water but is also reported as opportunistic human pathogen capable of causing nosocomial infections (Fernández et al., 2015). OTU42 was positively influenced by the eluate application at the all the dosages, particularly at 2 g L^−1^. It belongs to *Pseudomonas chlororaphis*, that is commonly found in soil and studied for its PGPR traits and biocontrol activity (Zhang et al., 2020; Heredia-Ponce et al., 2021). OTU103, was scarcely but positively influenced at the dosages of 4 and 40 g L^−1^. The OTU belongs to *Lysobacter capsici*, widely studied for its biocontrol activity against phytopathogens (Liu et al., 2019). Finally, OTU174 was positively influenced by the eluate application, and it belongs to *Acinetobacter johnsonii*, which is reported to be both an opportunistic pathogen rarely causing nosocomial infections (Montaña et al., 2016), as well as a PGPR in sugar beet (Shi et al., 2011). More analyses are required in order to understand if the eluate has a particular affinity with *A. johnsonii* and whether this could represent a risk.

Metastats analysis reports an applied on fungal OTUs important presence of many unclassified fungi that were positively influenced by the eluate dose of 40 g L^−1^. Other OTUs were taxonomically classified and belong to known PGPF such as *Mortierella elongata* (OTU8 and OTU20098), *Mortierella alpina* (OTU18), *Mortierella minutissima* (OTU75). *Mortierella* spp. are omnipresent soil-borne fungi, known to include endophytes and saprophytes species. Their PGP proprieties have been measured on different crops, they are reported to be phytohormones producers, to increase phosphorus solubilization, to be strong biocontrol agents, and to influence the carbon cycling in soil (Hanson et al., 2008). Moreover, it is reported to be capable of symbiosis with other endophytic bacteria (Liao, 2021; Ozimek and Hanaka, 2021). On the other hand, OTU6, OTU10, OTU79, and OTU10708 showed a reduction trend upon eluate application. These OTUs belong to known plant pathogens: *Fusarium equiseti*, *Fusarium oxysporum*, *Fusarium solani*, *Fusarium* sp. (Zhang et al., 2006; Michielse and Rep, 2009; Hami et al., 2021). Statistical analysis showed significative reduction only for OUT6 and OTU10.

Hence, it could be speculated that the eluate positively affects the bacterial population by “selecting” distinct genera or species, that mostly belong to PGPR/PGPF, and are therefore desirable in cultivated soils. More analyses are required in order to understand if the eluate has a particular affinity with the mentioned bacterial species.

## Conclusion

The present work aimed to measure the effect on soil proprieties of a candidate plant biostimulant obtained from the industrial production of Lactic Acid Bacteria, with the final goal to create added value for an industrial organic side-product, setting the frame for a wide-scale circular economy.

The study demonstrated that no toxic effect occurred on soil upon the eluate application. Eluate applications had a stronger impact on bacterial diversity which resulted to be lowered even at the lower eluate dosages, while fungi diversity increased along with higher dosages. Moreover, the eluate increased the rate of available K in soil, as well as the overall content of humic substances over the total organic fraction. Despite the general reduction observed in bacterial diversity with increasing concentration of the by-product, the relative abundance of some beneficial PGPR species are positively driven by the eluate application. In addition to this, it proved to be able to enhance the presence of beneficial fungi while negatively impacted the presence of the plant pathogen *Fusarium* spp. Taken together, these features outline the advantageous and eco-sustainable impact of the lactic eluate to potentially improve plant nutrition, soil fertility, and soil richness in positive Plant Growth Promoting Microorganisms. These properties will be beneficial when integrated in current agronomic practices, especially among cultivated soils that are highly exploited by intensive cultivation system, and where beneficial microorganisms could be having a significant and balancing impact.

## Data availability statement

The datasets presented in this study can be found in online repositories. The names of the repository/repositories and accession number(s) can be found at: https://www.ncbi.nlm.nih.gov/bioproject/PRJNA827989.

## Author contributions

GB: conceptualization, methodology, formal analysis, and writing—original draft. ET and MG: conceptualization, methodology, data analysis, and data interpretation. GMB: methodology and data analysis. CM, SR, FB, VT, AF, FC, RB, SS, and CS: methodology. PC: conceptualization, writing—review and editing, and supervision. FV: methodology and writing—review and editing the draft. EP: conceptualization, software, formal analysis, resources, writing—review and editing, supervision, and project administration. All authors contributed to the article and approved the submitted version.

## Conflict of interest

FV is employed by SACCO S.r.l., and RB, SS, and CS are employed by LANDLAB S.r.l.

The remaining authors declare that the research was conducted in the absence of any commercial or financial relationships that could be construed as a potential conflict of interest.

## Publisher’s note

All claims expressed in this article are solely those of the authors and do not necessarily represent those of their affiliated organizations, or those of the publisher, the editors and the reviewers. Any product that may be evaluated in this article, or claim that may be made by its manufacturer, is not guaranteed or endorsed by the publisher.

## Footnotes

^1^ http://hannonlab.cshl.edu/fastx_toolkit/

^2^ http://www.R-project.org/

## Supplementary materials

The Supplementary materials for this article can be found online at: https://www.frontiersin.org/articles/10.3389/fpls.2022.907349/full#supplementary-material
