# Supplementary material for: Agronomical valorization of eluates from the industrial production of microorganisms: Chemical, microbiological, and ecotoxicological assessment of a novel putative biostimulant
Source: Front Plant Sci. 2022 Jul 22;13:907349. doi: 10.3389/fpls.2022.907349 (PMC9356291; doi:10.3389/fpls.2022.907349)
Supplement: Supplementary file 1 [file Data_Sheet_1.docx]

Supplementary Material

# Supplementary Tables

Table 1. Eluate aminoacidic composition determined with HPLC.

| Aminoacid | Content (%) |
| --- | --- |
| Alanine | 0.96% |
| Arginine | 0.40% |
| Asparagine (incl. Aspartic acid) | 1.07% |
| Hydroxyproline | 0.25% |
| Cysteine | 0.24% |
| Glutamic acid | 1.83% |
| Glycine | 0.55% |
| Histidine | 0.20% |
| Isoleucine | 0.46% |
| Leucine | 0.74% |
| Lysine | 0.77% |
| Methionine | 0.13% |
| Phenylalanine | 0.41% |
| Proline | 0.45% |
| Serine | 0.46% |
| Threonine | 0.41% |
| Tryptophan | 0.08% |
| Tyrosine | 0.29% |
| Valine | 0.62% |

Table 2. OTU table with significantly different bacterial OTUs. Taxonomy was attributed using RDP and confirmed with the BLASTN function of NCBI.

| OTUs | BLASTN | S_ab score |
| --- | --- | --- |
| Otu 1 | *Arthrobacter globiformis* | 1.000 |
| Otu 2 | *Sphingomonas spp.* | 1.000 |
| Otu 3 | *Bacillus drentensis* | 1.000 |
| Otu 4 | *Priestia megaterium* | 1.000 |
| Otu 5 | *Pseudomonas sp.* | 1.000 |
| Otu 6 | *Laceyella spp.* | 0.997 |
| Otu 8 | *Bacillus litoralis* | 1.000 |
| Otu 14 | Uncultured bacterium | na |
| Otu 15 | Microvirga subterranea | 1.000 |
| Otu 17 | Uncultured bacterium | na |
| Otu 21 | Uncultured bacterium | na |
| Otu 22 | Uncultured bacterium | na |
| Otu 23 | *Sphingomonas spp.* | 0.982 |
| Otu 24 | uncultured *Firmicutes* | 0.978 |
| Otu 25 | *Pseudomonas putida* | 1.000 |
| Otu 28 | *Bacillus soli* | 0.998 |
| Otu 42 | *Pseudomonas chlororaphis* | 1.000 |
| Otu 45 | *Bhargavaea ginsengi* | 1.000 |
| Otu 52 | Uncultured bacterium | na |
| Otu 60 | *Paenisporosarcina quisquiliarum* | 1.000 |
| Otu 68 | *Adhaeribacter spp.* | 0.98 |
| Otu 70 | *Pontibacter populi* | 0.997 |
| Otu 83 | Uncultured bacterium | na |
| Otu 95 | *Pseudomonas resinovorans* | 1.000 |
| Otu 103 | *Lysobacter capsici* | 1.000 |
| Otu 155 | *Chthoniobacter sp.* | 0.981 |
| Otu 174 | *Acinetobacter johnsonii* | 1.000 |
| Otu 249 | *Salinimicrobium sp.* | 0.988 |
| Otu 285 | *Limnobacter thiooxidans* | 0.998 |
| Otu 323 | *Pontibacter amylolyticus* | 1.000 |
| Otu 328 | *Pontibacter populi* | 0.995 |
| Otu 383 | *Rheinheimera soli* | 0.986 |

Table 3. OTU table with significantly different fungal OTUs. Taxonomy was attributed using Mycobank database and confirmed with the BLASTN function of NCBI.

| OTUs | BLASTN | % ID |
| --- | --- | --- |
| Otu 6 | *Fusarium equiseti* | 98.95 |
| Otu 8 | *Mortierella elongata* | 100 |
| Otu 10 | *Fusarium oxysporum* | 100 |
| Otu 18 | *Mortierella alpina* | 100 |
| Otu 24 | Unidentified Fungi | n.a. |
| Otu 37 | Unidentified Fungi | n.a. |
| Otu 44 | Unidentified Fungi | n.a. |
| Otu 53 | Unidentified Fungi | n.a. |
| Otu 63 | Unidentified Fungi | n.a. |
| Otu 65 | Unidentified Fungi | n.a. |
| Otu 75 | *Mortierella minutissima* | 100 |
| Otu 79 | *Fusarium solani* | 100 |
| Otu 83 | Unidentified Fungi | n.a. |
| Otu 88 | Unidentified Fungi | n.a. |
| Otu 103 | Unidentified Fungi | n.a. |
| Otu 115 | Unidentified Fungi | n.a. |
| Otu 151 | Unidentified Fungi | n.a. |
| Otu 271 | Unidentified Fungi | n.a. |
| Otu 340 | Unidentified Fungi | n.a. |
| Otu 379 | Unidentified Fungi | n.a. |
| Otu 10708 | Fusarium sp. | 100 |
| Otu20098 | *Mortierella elongata* | 100 |
| Otu 24362 | Unidentified Fungi | n.a. |
| Otu 73732 | *Fusarium equiseti* | 97.89 |
| Otu 74618 | Unidentified Fungi | n.a. |
